# An Evaluation of Jansson’s Method to Deconvolve Overlapped Gas Chromatographic Peaks

**DOI:** 10.6028/jres.093.099

**Published:** 1988-06-01

**Authors:** Paul Benjamin Crilly

**Affiliations:** Hewlett-Packard Company, Box 900, Avondale, PA 19311

## Introduction

It has been reported [1,2] that Jansson’s Method [3] can be used to deconvolve severely overlapped gas chromatographic peaks. Initial testing [1,2] indicates the method will give improved performance over conventional graphical peak resolution techniques such as perpendicular drop and shoulder quantitation [4,5].

Jansson’s Method is an iterative non-linear algorithm that uses the prior knowledge of peak non-negativity and maximum peak height for an improved estimate of the true chromatogram [3]. The method only requires a knowledge of the instrument’s impulse response function and maximum peak height. Jansson’s Method does not require any prior information on how many peaks are overlapped.

This paper will show the results of a comprehensive evaluation of Jansson’s Method to resolve overlapped peaks that have been generated from two different sample mixtures. As a benchmark, these peaks were also resolved using shoulder quantitation and perpendicular drop techniques. As a second benchmark, these same sample mixtures were analyzed using an instrument with a relatively long column so that the peaks were fully resolved without using non-chemical peak resolving methods.

## Theory

A gas chromatographic process can be modeled as follows [1]

g=h∗x+n (1)

where *g* is the observed peaks (raw data), *h* is the system impulse response function, *x* is the true peak shape, *n* is random noise and * is the convolution operator. Variables *g*, *h, x*, and n are all functions of time. It is assumed the system is time invariant. The convolution of functions *x* and *h* can cause the perfectly resolved peaks in *x* to become severely overlapped.

Jansson’s Method can be used to obtain an estimate of the true peak shape, *x*, (given functions *g* and *h*) as follows [3]

$${\hat{x}}^{k+1}={\hat{x}}^{k}+r\left\{{\hat{x}}^{k}\right\}\left[g-h*{\hat{x}}^{k}\right]$$ (2a)

with

$$r\left\{{\hat{x}}^{k}\right\}=b\left(1-2/c\left|{\hat{x}}^{k}-c/2\right|\right)$$ (2b)

where ${\hat{x}}^{k}$ is the *k*’th estimate of the true peak shape *x*, *b* is the relaxation constant, and *c* is the maximum peak amplitude. Relaxation function $r\left\{{\hat{x}}^{k}\right\}$ constrains the estimate to within its physical limits of 0 and *c*.

## Experimental Procedure

The chromatographic analysis was done on a Hewlett-Packard 5890A Gas Chromatograph with a thermal conductivity detector. Two mixtures consisting of 50/50 and 10/90 concentrations of ethyl benzene and m-xylene samples were used to generate the chromatograms. Each sample was injected and analyzed 20 times so there would be a reasonable statistical basis for the goodness of any one method.

**Figure 1:**
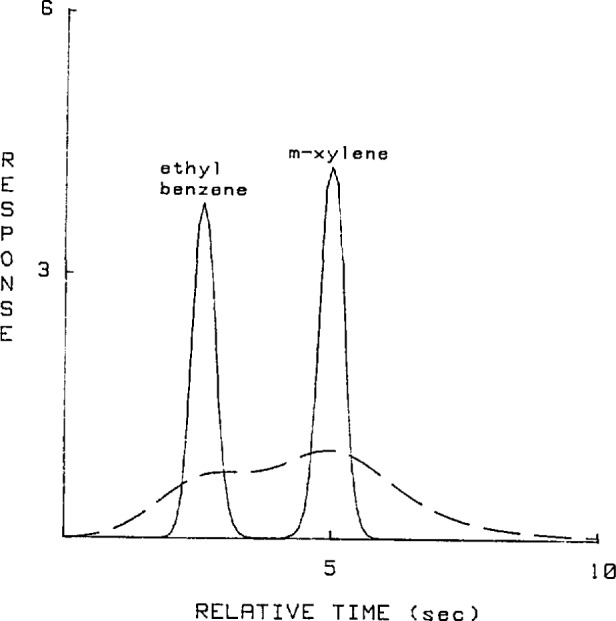
Solid line plot is the chromatogram of the 50/50 mixture of ethyl benzene and m-xylene deconvolved using Jansson’s Method with 160 iterations. Dotted line plot is the original chromatogram [2].

**Figure 2:**
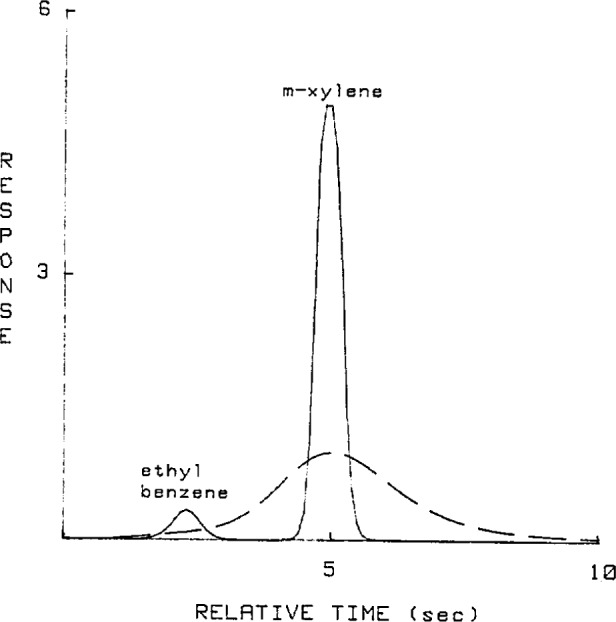
Solid line plot is the chromatogram of the 10/90 mixture of ethyl benzene and m-xylene deconvolved using Jansson’s Method with 160 iterations. Dotted line plot is the original chromatogram [2].

An instrument with a relatively short capillary column (10 meter) generated overlapped chromatograms whose resolution was 0.42 and are shown in the dotted line plots of figures 1 and 2. These overlapped peaks were resolved using Jansson’s Method, shoulder quantitation and perpendicular drop techniques. The estimates obtained using Jansson’s Method after 160 iterations are shown in the solid line plots of figures 1 and 2. Another set of chromatograms was generated using an instrument with a longer (50 meter) and narrower capillary column so that the peaks generated were fully resolved. For each method, the relative errors, peak quantity variances and relative retention time variances were calculated and are presented in tables 1 to 3.

## Results and Discussion

As table 1 indicates, the quantitation accuracy of peaks resolved using Jansson’s Method is similar to what can be obtained using an instrument with a relatively long column and is about an order of magnitude better than the graphical methods. The peak quantitation accuracies for the graphical methods are similar to what Mikkelson et al. [4] and Altmayer [5] have reported. The peak quantity and relative retention time variances reported in tables 2 and 3 indicate Jansson’s Method compares favorably to the long column method and is better than the graphical methods.

It should be noted that the overlapped peaks of figures 1 and 2 were overlapped to such a degree that it was almost impossible to implement the graphical methods since it was difficult to locate the peaks and valleys.

For a peak resolution of 0.42, a 10 Hz sample rate and pre/post smoothing using a nine point polynomial filter, Jansson’s Method could deconvolve data whose signal-to-noise ratio was as low as 60:1. However, the signal-to-noise performance of Jansson’s Method will depend on the noise power spectral density, degree of peak overlap, data system sampling rate and whether the data is pre and/or post smoothed. To a lesser extent, the signal-to-noise performance will depend on the number of iterations and the relaxation constant chosen.

In conclusion, Jansson’s Method can be used to extend the capabilities of an instrument and provide faster analysis time. The initial results have been encouraging such that further research is justified.

## Figures and Tables

**Table 1:** Relative error comparison for various peak resolving methods [2]

	Relative Error			
Sample concentration (%)	Long column^a^ (%)	Jansson’s Method^b^,^c^ (%)	Shoulder quantitation^c^,^d^ (%)	Perpendicular drop^c^,^d^ (%)
ethyl benzene: 50	−0.04	+0.08	− 6.36	−35.35
m-xylene: 50	+0.04	−0.07	+6.07	+33.74
ethyl benzene: 10	+2.01	−4.68	−33.26	−78.67
m-xylene: 90	−0.20	+0.46	+3.27	+7.72

a Peaks were fully resolved without using non-chemical techniques.

b 160 iterations used.

c Original convolved peak had a resolution of 0.42.

d Since the peaks were so severely overlapped, implementing this method required prior knowledge that two peaks were present and their approximate retention times.

**Table 2:** Peak quantity variances for various peak resolving methods [2]

	Peak Quantity Variance^a^			
Sample concentration(%)	Long column	Jansson’s Method^b^	Shoulder quantitation	Perpendicular drop
ethyl benzene: 50	0.26	0.31	0.23	10.09
m-xylene: 50	0.25	0.29	0.19	4.66
ethyl benzene: 10	8.63	3.39	11.60	19.05
m-xylene: 90	0.87	0.32	0.73	0.37

a Units are in percent standard deviation.

b 160 iterations used.

**Table 3:** Relative retention time variance for various peak resolving methods [2]

Relative Retention Time Variance^a^				
Sample concentration (%)	Long column	Jansson’s Method^b^	Shoulder quantitation	Perpendiccular drop
ethyl benzene: 50				
	0.31	0.93	4.46	4.46
m-xylene: 50				
ethyl benzene: 10				
	4.33	2.57	3.54	3.54
na-xylene: 90				

a Units are in percent standard deviation.

b 160 iterations used.
